# Flecainide exerts paradoxical effects on sodium currents and atrial arrhythmia in murine *RyR2-P2328S* hearts

**DOI:** 10.1111/apha.12505

**Published:** 2015-04-23

**Authors:** S C Salvage, J H King, K H Chandrasekharan, D I G Jafferji, L Guzadhur, H R Matthews, C L-H Huang, J A Fraser

**Affiliations:** 1 Physiological Laboratory, University of CambridgeCambridge, UK; 2 Department of Biochemistry, University of CambridgeCambridge, UK

**Keywords:** atrial arrhythmia, conduction velocity, CPVT, flecainide, Na^+^ currents, ryanodine receptor

## Abstract

**Aims:**

Cardiac ryanodine receptor mutations are associated with catecholaminergic polymorphic ventricular tachycardia (CPVT), and some, including *RyR2-P2328S*, also predispose to atrial fibrillation. Recent work associates reduced atrial Na_v_1.5 currents in homozygous *RyR2-P2328S* (*RyR2*^S/S^) mice with slowed conduction and increased arrhythmogenicity. Yet clinically, and in murine models, the Na_v_1.5 blocker flecainide reduces ventricular arrhythmogenicity in CPVT. We aimed to determine whether, and how, flecainide influences atrial arrhythmogenicity in *RyR2*^S/S^ mice and their wild-type (WT) littermates.

**Methods:**

We explored effects of 1 *μ*m flecainide on *WT* and *RyR2*^S/S^ atria. Arrhythmic incidence, action potential (AP) conduction velocity (CV), atrial effective refractory period (AERP) and AP wavelength (*λ* = CV × AERP) were measured using multi-electrode array recordings in Langendorff-perfused hearts; Na^+^ currents (*I*_Na_) were recorded using loose patch clamping of superfused atria.

**Results:**

*RyR2*^*S/S*^ showed more frequent atrial arrhythmias, slower CV, reduced *I*_Na_ and unchanged AERP compared to *WT*. Flecainide was anti-arrhythmic in *RyR2*^S/S^ but pro-arrhythmic in WT. It increased *I*_Na_ in *RyR2*^S/S^ atria, whereas it reduced *I*_Na_ as expected in *WT*. It increased AERP while sparing CV in *RyR2*^*S/S*^, but reduced CV while sparing AERP in *WT*. Thus, *RyR2*^*S/S*^ hearts have low *λ* relative to *WT*; flecainide then increases *λ* in *RyR2*^*S/S*^ but decreases *λ* in *WT*.

**Conclusions:**

Flecainide (1 *μ*m) rescues the *RyR2-P2328S* atrial arrhythmogenic phenotype by restoring compromised *I*_Na_ and *λ*, changes recently attributed to increased sarcoplasmic reticular Ca^2+^ release. This contrasts with the increased arrhythmic incidence and reduced *I*_Na_ and *λ* with flecainide in WT.

Atrial fibrillation (AF) is the most common sustained arrhythmia, predisposing to significant clinical morbidity and mortality (Benjamin *et al*. 1998, Stewart *et al*. 2002, Davis *et al*. 2012), yet its physiological mechanisms are incompletely understood. Nevertheless, acute atrial arrhythmogenesis may be related not only to cellular Ca^2+^ homeostasis but also to altered action potential (AP) conduction and recovery (Zhang *et al*. 2011, King *et al*. 2013c).

Abnormal Ca^2+^ release can arise from cardiac ryanodine receptor-2 (*RyR2*) mutations or a loss of calsequestrin-2 (*CSQ2*) (Priori & Chen 2011), potentially providing arrhythmic triggers (Mackenzie *et al*. 2001, 2004, Bootman *et al*. 2006, Zhang *et al*. 2010), thereby leading to catecholaminergic polymorphic ventricular tachycardia (CPVT) (Priori & Chen 2011, Zhang *et al*. 2013a). Certain *RyR2* mutations are also associated with AF (Bhuiyan *et al*. 2007, Sumitomo *et al*. 2007). The *RyR2-P2328S* mutation is associated with high incidences of both CPVT and atrial tachycardia (AT), despite normal cardiac structure (Swan *et al*. 1999, Laitinen *et al*. 2001). Murine hearts with a homozygotic *RyR2-P2328S* (*RyR2*^S/S^) mutation demonstrate both atrial and ventricular arrhythmic tendencies (Goddard *et al*. 2008, Zhang *et al*. 2011, 2013b, King *et al*. 2013b,c) providing a useful experimental model. Atrial *RyR2*^S/S^ myocytes show diastolic elevations in intracellular [Ca^2+^] attributed to increased SR Ca^2+^ release (Zhang *et al*. 2011). This would be expected to increase Na^+^/Ca^2+^ exchange (NCX) activity, accounting for delayed afterdepolarizations (DADs) causing triggered activity, implicated in the arrhythmic phenotype (King *et al*. 2013c).

It has recently been reported that flecainide exerts anti-arrhythmic effects in human CPVT (Watanabe *et al*. 2009, van der Werf *et al*. 2011). Flecainide reduced bigeminy and biventricular tachycardia, ECG features associated with human CPVT, in murine *CSQ2*^−/−^ hearts. However, there is debate over the anti-arrhythmic mechanism of flecainide in CPVT. It has been suggested that flecainide directly reduces both RyR2-mediated Ca^2+^ release and the consequent triggering events (Watanabe *et al*. 2009, Hilliard *et al*. 2010, Hwang *et al*. 2011). Alternatively, anti-arrhythmic actions of flecainide may be attributed to inhibition of Na_v_1.5 function, thereby decreasing membrane excitability and the likelihood of triggered activity (Liu *et al*. 2011).

Further questions concerning the anti-arrhythmic mechanism of flecainide arise from reports implicating reduced conduction velocity (CV) in *RyR2*^S/S^ atria relative to *WT*. These reports show that the impaired CV is secondary to reduced *I*_Na_ rather than abnormal fibrosis or structural remodelling (King *et al*. 2013b,c). Reduced CV has also been shown with other mutations associated with diastolic Ca^2+^ release and murine atrial arrhythmias including *CREM*-IbΔC-X (Li *et al*. 2014) and *CSQ2*^*−/−*^ (Glukhov *et al*. 2013). In each case, the resultant reduced AP wavelength (*λ*) would increase the likelihood of re-entrant arrhythmias (King *et al*. 2013a).

Na_v_1.5 inhibition by flecainide might be expected to further reduce CV and *λ* in *RyR2*^*S/S*^ atria. Yet, Na_v_1.5 inhibition and consequent reduced Na^+^ entry might also increase forward-mode NCX activity (Liu *et al*. 2011, Sikkel *et al*. 2013), thus reducing diastolic Ca^2+^. Flecainide has also been shown to reduce RyR2-mediated Ca^2+^ release (Watanabe *et al*. 2009, Hilliard *et al*. 2010, Hwang *et al*. 2011). This study sought to assess whether, at the tissue level, there was a reduced arrhythmic tendency in the presence of flecainide in a system showing a RyR2 abnormality accompanied by compromised Na^+^ channel function and AP conduction velocity. We then investigated the alterations in arrhythmic tendency brought about by flecainide through an assessment of Na^+^ channel function, conduction velocity and recovery characteristics that might together rescue *λ*, otherwise compromised by the *RyR2*^S/S^ mutation. This would establish a tissue-level significance of the previous cellular level results suggesting that altered Ca^2+^ homeostasis could affect Na^+^ channel function.

The experiments therefore test the influence of flecainide on arrhythmogenicity in *RyR2*^*S/S*^ and *WT* atria and correlate this with its influence on *I*_Na_, CV, AERP and *λ*. We thus complement a recent study reporting similar anti-arrhythmic inhibitory actions of another class Ic anti-arrhythmic agent, propafenone, on Ca^2+^ release events during atrial fibrillation in a *CSQ2*^−/−^ model of CPVT (Faggioni *et al*. 2014), although *I*_Na_ and CV were not measured in that latter study.

## Materials and methods

### Experimental animals

All procedures were performed in licensed institutional premises under a UK Home Office project licence approved by a university ethics review board, under the UK Animals (Scientific Procedures) Act (1986), and conforming to European Parliament Directive 2010/63/EU. 3.5- to 11.5-month-old wild-type (*WT*, *n* = 22) and *RyR2-P2328S* (*RyR2*^S/S^, *n* = 23) inbred 129/Sv mice (Harlan, UK) were kept in plastic cages at room temperature in 12-h light/dark cycles. Mice had free access to sterile rodent chow and water. All chemical agents were purchased from Sigma-Aldrich (Poole, UK) except where otherwise indicated, with effects of flecainide studied at concentrations of 1 and 5 *μ*m and dantrolene Na at 10 *μ*m.

### Experimental set-up in isolated Langendorff-perfused hearts

Mice were killed by cervical dislocation (Schedule 1: UK Animals (Scientific Procedures) Act 1986). Hearts were excised and placed in ice-cold bicarbonate-buffered Krebs-Henseleit solution (KH) containing (mm) NaCl 119, NaHCO_3_ 25, KCl 4, KH_2_PO_4_ 1.2, MgCl_2_ 1, CaCl_2_ 1.8, glucose 10 and Na-pyruvate 2; pH 7.4, 95% O_2_/5% CO_2_ (British Oxygen Company, Manchester, UK), then cannulated and perfused with KH as previously described (Zhang *et al*. 2010, 2011). After a 10- to 15-min stabilization period, hearts were paced using an Ag/AgCl electrode at the epicardial surface of the right atrium. First, a regular pacing protocol imposed successive trains of 100 stimuli at frequencies of 5, 6.67, 8 and 10 Hz respectively. This was followed by a programmed electrical stimulation (PES) protocol which first paced at 10 Hz for 20 s. It then applied drive trains consisting of cycles of eight paced stimuli (S1), each followed by a single extra stimulus (S2). The S1–S2 interval was initially equal to the pacing interval, then reduced by 1 ms with each subsequent cycle until S1–S2 = 6 ms. Both the *WT* and *RyR2*^S/S^ hearts were stimulated using square-wave stimuli of 2 ms duration and amplitudes of twice diastolic excitation threshold (Sabir *et al*. 2007) (DS2A isolated constant voltage stimulator; Digitimer, Welwyn Garden City, Herts., UK). There was no significant difference in mean excitation threshold between *WT* and *RyR2*^S/S^ hearts [thresholds: *WT*, 1.68 ± 0.37 V (*n* = 9); *RyR2*^S/S^, 1.75 ± 0.39 V (*n* = 17); *P* = 0.69]. This protocol provided both arrhythmic incidences, defined as an occurrence of two or more non-stimulated atrial electrograms, and AERPs, defined as the period when the cell is refractory to the initiation of new APs, such that no atrial electrogram results from the S2 stimuli.

### Multi-electrode array recordings and conduction velocity vector analysis

**Figure 1:**
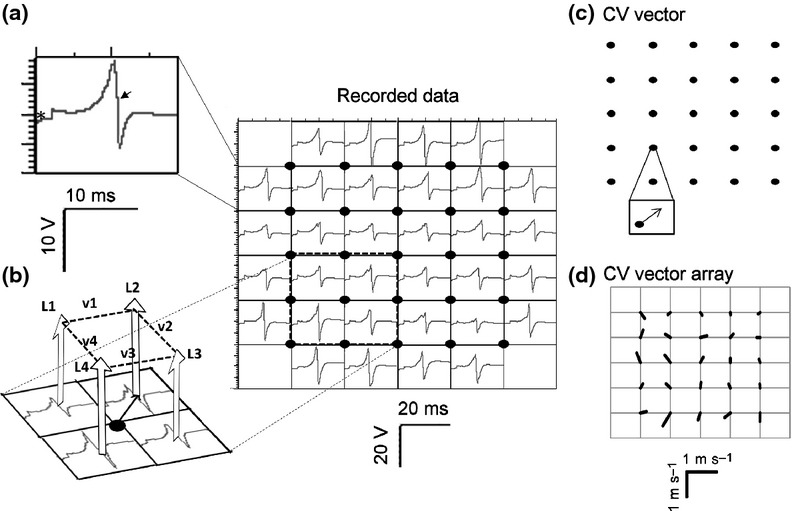
Conduction velocity analysis. A representative MEA recording is displayed as a set of individual traces obtained at each electrode site in the centre panel. Panels (a–d) illustrate the data analysis in which (a) the local activation time (LAT) is determined from the maximum negative d*V*/d*t* (arrowhead) of atrial electrograms at each recording site. (b) LATs from four neighbouring recording sites are used to derive (c) the conduction velocity vector for each 2 × 2 square of electrodes and then visually inspected (d) to ensure the absence of wavefront collision/splitting.

Multi-electrode array (MEA) recordings were made from the epicardial LA surface of both *WT* and *RyR2*^*S/S*^ hearts during stimulation protocols. Each MEA (ME32-FAI-System; Scientifica, Uckfield, UK) contained 32 recording electrodes of diameter 50 *μ*m that were arranged in an array of successive rows of 4, 6, 6, 6, 6 and 4 electrodes within a 1.5 × 1.5 mm configuration with a 300-*μ*m interelectrode distance as shown in Figure1. Data were sampled at 10 kHz per channel. The positions of the stimulating electrode and the MEA were consistent throughout each experiment.

Local activation times (LATs) were calculated as the time from stimulation to the maximum negative rate of voltage change, (d*V*/d*t*)_max_ of the extracellular atrial electrogram recording of the AP. The maximum negative deflection is a consistently identifiable feature of the waveform, corresponding to the intracellular AP peak, which has previously been employed to assess relative arrival times in extracellular multi-electrode recordings (Lambiase *et al*. 2009, Zhang *et al*. 2014). It was then possible to determine the median LAT for each atrial electrogram (Fig.1A). Relative LATs were then determined by subtracting the median LAT from the individual LATs for each atrial electrogram. Finally, the median relative LAT was found for each recording electrode over all the atrial electrograms at each recording frequency.

A velocity vector was calculated and attributed to the centre of these sites (Fig.1B). Column (*y*) and row (*x*) time vector components were calculated from the median atrial electrogram LATs at four neighbouring recording sites (L1 to L4, ms) as *y* = ((L1 + L2) − (L3 + L4))/2 and *x* = ((L1 + L4) − (L2 + L3))/2 respectively. Velocity vector direction was calculated as *θ* = atan2(*x*/0.3, *y*/0.3), and its magnitude (mm ms^−1^) was calculated as


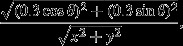


where 0.3 is the electrode spacing in mm.

This was done for every interleaved combination of neighbouring recording sites, producing a 5 × 5 grid of CV vectors each spaced 0.3 mm apart (Fig.1C). The vectors were then plotted and inspected for wave collisions and wave breaks that would break the necessary assumption of uniform conduction direction between adjacent pins (Fig.1D); any such vectors were manually removed. Median velocity and standard deviation were calculated from the remaining vectors, yielding a single value of CV under each intervention and pacing rate for each of the hearts studied in each experimental group. *λ* was subsequently calculated in hearts for which both CV and AERP values were available (0 and 1 *μ*m flecainide).

### Loose patch-clamp recording and assessment of Na^+^ current

Loose patch-clamp experiments were performed as previously described (King *et al*. 2013b). This technique was chosen to permit measurement of Na^+^ currents in whole, perfused, freshly dissected atria, without the potential disruption of intracellular Ca^2+^ homeostasis that might occur during cell isolation and preparation for a tight patch approach. The maintenance of intercellular connectivity allowed recording of Na^+^ currents under similar experimental conditions to those employed in the CV experiments. Na^+^ currents recorded from such experiments have been shown to be in agreement with those obtained from tight patch techniques (Eickhorn *et al*. 1990). Micropipettes were pulled from plain thick-walled borosilicate glass capillary (GC 150-10; Harvard Apparatus, Kent, UK) using a micropipette puller (Brown-Flaming Model P-97, Sutter Instrument Company, Novato, CA, USA). The pipette was held in a micromanipulator mounted on the stage of a compound microscope and scribed transversely at a point along its shaft where it was a little over 40 *μ*m in diameter using a diamond knife under visual control at 250 × magnification. Transverse force applied to the distal tip caused the pipette to fracture at this point orthogonal to its axis. The pipette was then fire-polished using an electrically heated nichrome filament at 400 × magnification to produce a tip with an internal diameter of approx. 40 *μ*m, previously shown to yield the most consistent Na^+^ currents in atrial patches (King *et al*. 2013b). Internal tip diameters were measured at 1000 × magnification using a calibrated eyepiece graticule. Pipettes were bent through an angle of about 45° approx. 1 mm from the tip, so that it approached the membrane vertically when mounted on the headstage of the recording amplifier.

The left atrium was mounted upon a Sylgard (Dow Chemical Company, Chicago, IL, USA) gel platform and placed in an actively grounded bath filled with KH buffer maintained at just above room temperature (25 ± 3 °C) using a heat exchanger and fluid circulator. The pipette was filled to two-thirds along its shaft with KH buffer; an air-filled line connected to the pipette holder allowed suction to be applied during loose patch formation. Electrical connections to bath and pipette were made with Ag/AgCl electrodes. Loose patch-clamp recordings were carried out using a custom-built amplifier designed to compensate for leakage current, series resistance errors and pipette capacitance (Stühmer & Almers 1982). The pipette was lowered until a resistance increase was observed, indicating contact with the atrial surface. Gentle suction was then applied to draw a patch of membrane into the pipette tip. Voltage-clamp steps were delivered under computer control; a negative-going clamp step represents a corresponding depolarization relative to the resting membrane potential.

Activation properties were investigated with a series of depolarizing test pulses of 75 ms duration, delivered 5 ms following the beginning of the sampling period using a P/4 pulse protocol (Bezanilla & Armstrong 1977). Although the P/4 protocol corrects for relative errors during the clamp step itself, it also adds baseline offsets during the correction procedure. The underlying drift in clamp voltage was <1 mV in all cases. The test voltage steps delivered single depolarizing voltage excursions from rest ranging from 20 mV to 120 mV, incremented by 10 mV between trials. The complete series of trials was bracketed by 80 mV depolarizing pulses to check patch stability. The depolarizing voltage steps elicited distinct inward currents which activated rapidly and then inactivated, thus closely resembling previous loose patch-clamp measurements (Almers *et al*. 1983, Roberts *et al*. 1986). These were often followed by increasing outward currents; nevertheless, the time course of peak *I*_Na_ remained clearly separable permitting an assessment of Na^+^ channel expression and activation. In addition, some activation experiments were performed in the presence of 10 *μ*m dantrolene Na, as a specific RyR blocker, at the 80 mV voltage step used for testing patch stability. Inactivation properties were investigated by incorporating a depolarizing pre-pulse of 5 ms duration and variable amplitude immediately prior to the test pulse, which had a fixed magnitude of 100 mV from rest and a duration of 70 ms.

Clamp currents were filtered over the bandwidth DC-10 kHz (8-pole Bessel filter) and digitized at 50 kHz using custom written software. The resulting traces were not zeroed to the initial baseline to best display currents at each voltage step. Resting potential measurements were performed in a similar superfused atrial preparation using KCl-filled 10–20 MΩ glass microelectrodes in the isolated right atrial portions of the same hearts from which the left atria were obtained.

Variations in *I*_Na_ were measured with the loose patch-clamp method before and following alterations in the solution bathing the external face of the membrane. These involved raising the patch pipette, exchanging the solution in the bath and then re-establishing the patch at the same membrane location. *I*_Na_ records obtained in response to progressively increasing depolarizing steps from the resting potential gave virtually superimposable and thus reproducible current–voltage relationships before and following withdrawal and restoration of the patch pipette in a *WT* left atrium. Similar electrode withdrawals and reapplications during which external [Na^+^] was first reduced from 146 to 39 mm and then returned to 146 mm produced fully reversible reductions in the observed currents. The kinetics of these inward currents were similar to those described previously (Lemoine *et al*. 2011, King *et al*. 2013b) consistent with their representing *I*_Na_. The resting membrane potential measurements obtained independently using sharp intracellular microelectrodes from *n* = 15 or 16 cells of 3 or 4 hearts in the presence and absence of flecainide ranged from −70.43 ± 0.51 mV to −72.49 ± 0.56 mV (Table 1), a variation which was not significantly different and is within the error of tip potential recordings (Adrian 1956). This represented a consistent baseline voltage from which voltage excursions (V) could be imposed.

**Table 1:** Effects of flecainide in *WT* and *RyR2*^S/S^ atria

Genotype, flecainide (*μ*m)	*WT*, 0	*WT*, 1	*WT*, 5	*RyR*^*S/S*^_,_ 0	*RyR*^*S/S*^_,_ 1	*RyR*^*S/S*^_,_ 5
Arrhythmic incidence (mean events per heart)	1.1 ± 0.22, 10	2.56 ± 0.453, 9*	N/A	2.65 ± 0.377, 17†	1.59 ± 0.385, 17*	N/A
*I*_Na_ activation properties						
*I*_Na,max_ (nA)	−14.56 ± 0.30, 7	−9.62 ± 0.21, 7***	7.98‡ ± 1.94, 7	−9.29 ± 0.51, 6^†††^	−12.14 ± 0.31, 6**^†††^	−9.81 ± 0.90, 6
*k* (mV*)*	19.15 ± 1.32, 7	14.69 ± 1.49, 7	‡	13.15 ± 5.07, 6	11.97 ± 1.98, 6	26.3 ± 6.7, 6
V* (mV)	65.86 ± 1.06, 7	59.12 ± 1.50, 7*	‡	43.48 ± 7.51, 6†	48.42 ± 2.50, 6^††^	61.44 ± 4.98, 6
*I*_Na_ inactivation properties						
*I*_Na,max_ (nA)	−11.51 ± 0.15, 7	−8.48 ± 0.06, 7***	−6.53 ± 0.37, 7***	−9.58 ± 0.15, 6^†††^	−12.01 ± 0.20, 6***^†††^	−7.7 ± 0.1, 6***†
*k* (mV*)*	16.51 ± 0.83, 7	14.76 ± 0.44, 7	24.24 ± 3.75, 7	12.96 ± 0.81, 6†	14.58 ± 0.97, 6	16.4 ± 0.82, 6*
V* (mV)	56.69 ± 0.75, 7	56.69 ± 0.41, 7	53.1 ± 2.51, 7	49.98 ± 0.76, 6^†††^	53.64 ± 0.88, 6*†	53.88 ± 0.71, 6**
Resting potential (mV)	−70.92 ± 0.65, 16	−72.49 ± 0.56, 15	−70.43 ± 0.51, 16	−71.89 ± 0.51, 15	−70.67 ± 0.58, 15	−71.44 ± 0.28, 15
Conduction Velocity						
At 6 Hz (m s^−1^)	0.326 ± 0.018, 9	0.216 ± 0.019, 9***	0.168 ± 0.012, 8***	0.313 ± 0.026, 9	0.306 ± 0.018, 10^†††^	0.22 ± 0.22, 9**
At 8 Hz (m s^−1^)	0.381 ± 0.024, 15	0.216 ± 0.018, 10***	0.143 ± 0.016, 7***	0.275 ± 0.021, 14^†††^	0.279 ± 0.019, 10	0.188 ± 0.02, 9*
At 10 Hz (m s^−1^)	0.332 ± 0.020, 10	0.215 ± 0.021, 9**	0.164 ± 0.008, 4***	0.263 ± 0.026, 11	0.275 ± 0.020, 9	0.17 ± 0.21, 7*
AERP (ms)	24.56 ± 1.47, 9	27.67 ± 2.957, 9	N/A	23 ± 1.77, 17	34 ± 3.9, 17**	N/A
Wavelength (cm)	0.869 ± 0.072, 8	0.672 ± 0.086, 8*	N/A	0.627 ± 0.078, 10	0.738 ± 0.083, 10*	N/A

All results shown are means ±SEMs, sample size (*n*). The sample size indicates the number of hearts used for arrhythmic incidence, conduction velocity, AERP, wavelength, *I*_Na_ activation properties and *I*_Na_ inactivation properties. For resting potential, the sample size indicates the number of patches.

N/A, unavailable data due to loss of excitability. AERP, atrial effective refractory period; *k*, steepness factor describing the currents’ dependence upon voltage; V*, half-maximal voltage describing the voltage excursion corresponding to half-maximal current.

* Significant effects of 1 or 5 *μ*m flecainide compared to 0 *μ*m.

† Differences between *RyR2*^S/S^ and *WT* genotypes at the same flecainide concentration. Single, double and triple symbols denote *P* < 0.05, *P* < 0.01 and *P* < 0.001 respectively.

‡ A maximal recorded value, or unavailable data, where a Boltzmann function did not fit.

### Statistical analysis

Data are expressed as means ± SEM. Different experimental genotype groups of unpaired data were compared using two-way anova followed by Bonferroni-corrected *t*-tests if significant differences were found (graphpad prism v.6; GraphPad Software, Inc., La Jolla, CA, USA). Twenty-two *WT* and 26 *RyR2*^*S/S*^ hearts were used in the experiments – 7 *WT* and 6 *RyR*^*S/S*^ for the loose patch experiments, with the remainder paced at 6.67, 8 and 10 Hz and then taken through the PES protocol, allowing the determination of CV, arrhythmogenicity, AERP and wavelength in the same hearts when all protocols were successfully completed; ‘*n*’ denotes the number of hearts successfully studied in each case. Statistical significance was defined at *P <* 0.05.

## Results

The experiments determined the arrhythmic incidence during electrical pacing in both *RyR2*^S/S^ and *WT* hearts in the presence and absence of 1 and 5 *μ*m flecainide. These were then correlated with *I*_Na_, CV, AERP and *λ*, reductions which have been previously associated with arrhythmic substrate.

### Flecainide increases arrhythmic incidence in WT atria yet is anti-arrhythmic in RyR2^S/S^

**Figure 2:**
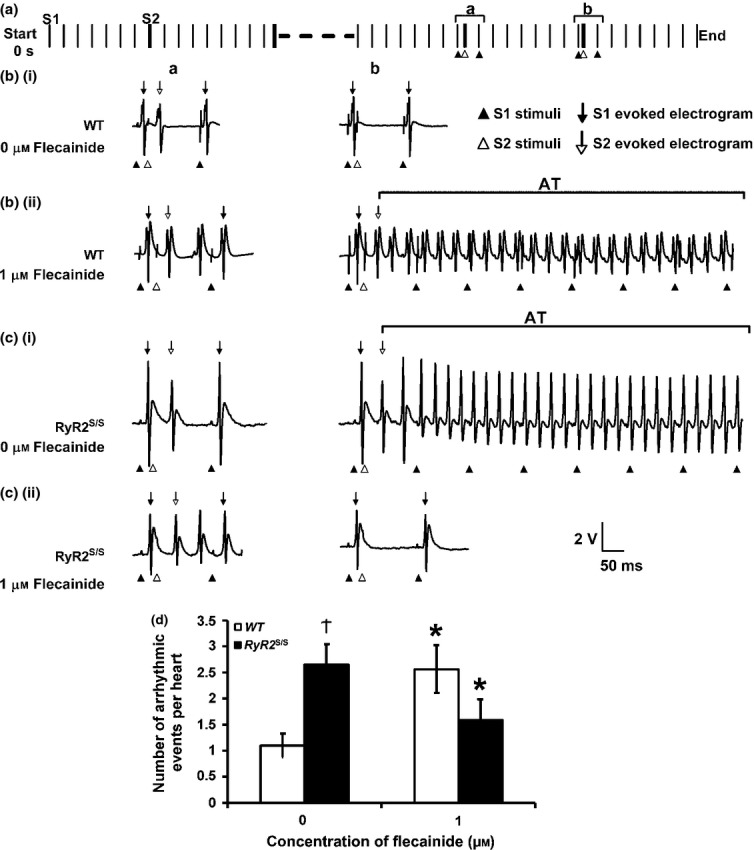
Contrasting actions of flecainide on arrhythmic incidence in *RyR2*^S/S^ and *WT*. (a) Illustration of the S1S2 stimulation protocol, consisting of repeated cycles of 8 S1 stimuli, each followed by a single extrasystolic S2 stimulus imposed at successively shorter S1S2 intervals. The first and last few cycles of the protocol are shown, with the intervening cycles omitted (dashed lines). The protocol was terminated when an S2 *either* failed to elicit an AP, as observed by a missing atrial electrogram, *or* produced an arrhythmia. Thus, panel (a) depicts the penultimate stimulus cycle, whose S2 stimulus successfully elicited conducting electrical activity (a), followed by the final cycle that induced either arrhythmia or refractoriness. Typical traces obtained from (b) *WT* and (c) *RyR2*^S/S^ before (i) and following (ii) introduction of 1 *μ*m flecainide were obtained from the last stimulus cycle whose S2 stimulus successfully elicited electrical activity (left panels) and the final cycle which induced either arrhythmia or refractoriness (right panels) as described above. The filled arrowheads indicate timings of regular (S1) stimulation, and the filled arrows indicate the resulting S1 atrial electrogram. The open arrowheads indicate the timing of the extrasystolic (S2) stimuli, and the open arrows indicate the resulting S2 atrial electrogram. The arrowheads are directly below the stimulus artefact, and the arrows are directly above the resulting atrial electrogram. Note that atrial electrogram conduction from the point of stimulation to the point of recording is slow relative to conduction of the stimulus artefact, such that the S2 stimulus artefacts can appear within the preceding S1 waveform at the recording site despite occurring after the atrial electrogram at the stimulus site. Panel (d) depicts the results of applying the PES protocol to 10 *WT* and 17 *RyR2*^*S/S*^ hearts to assess the incidence of arrhythmic events normalized to the number of hearts studied in each group. * denotes a difference (*P* < 0.05) at 0 and 1 *μ*m flecainide within a genotype. ^†^ denotes a difference (*P* < 0.05) between *RyR2*^S/S^ and *WT* genotypes at the same flecainide concentration.

As summarized in Table 1, incidences of atrial arrhythmia, classified as an occurrence of two or more non-stimulated atrial electrograms, were greater in untreated *RyR2*^S/S^ than *WT* as previously reported (King *et al*. 2013c). Flecainide (1 *μ*m) exerted anti-arrhythmic effects in *RyR2*^S/S^ in contrast to pro-arrhythmic effects in *WT* (Fig.2). Two-way anova demonstrated strong interactions (*P =* 0.0032; *F* = 9.644) between the effects of flecainide and genotype upon arrhythmic incidence. *Post hoc* testing demonstrated that *RyR2*^*S/S*^ hearts showed significantly higher incidences of arrhythmia than *WT* before flecainide challenge (*t* = 3.018; *P* < 0.05). Application of 1 *μ*m flecainide significantly increased arrhythmic incidence in *WT* (*t* = 2.584; *P* < 0.05) but significantly decreased it in *RyR2*^S/S^ (*t* = 2.520; *P* < 0.05). Following application of 1 *μ*m flecainide to both genotypes, there was no significant difference in arrhythmic incidence between *WT* and *RyR2*^S*/*S^ (*t* = 1.826; *P* > 0.05). Five micromolar flecainide was also tested; however, over a third of hearts (10 of 27) then became unresponsive to stimulation during either or both regular pacing (particularly at the higher frequencies) and PES.

### Flecainide increases Na^+^ currents in RyR2^S/S^ in contrast to decreasing Na^+^ currents in WT

**Figure 3:**
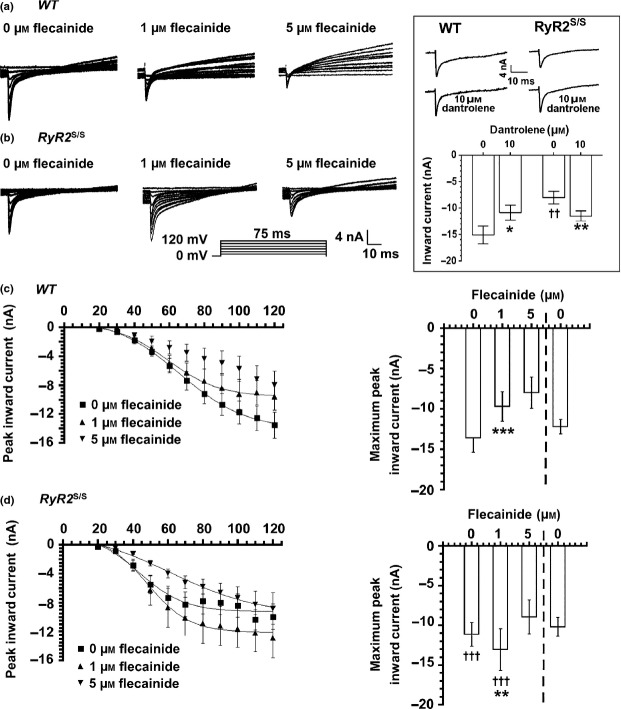
Paradoxical actions of flecainide on *I*_Na_ activation in *RyR2*^S/S^ and *WTatria*. Currents in response to depolarizing steps increased in 10 mV increments from 20 to 120 mV in voltage-clamped *WT* (a, *n* = 7) and *RyR2*^*S/S*^ (b, *n* = 6) left atria in the presence of 0, 1 and 5 *μ*m flecainide. Currents in response to an 80 mV depolarizing step under control conditions and in the presence of the specific RyR blocker dantrolene (10 *μ*m) are shown in the inset. The current–voltage relationships were fitted to Boltzmann functions for *WT* (c, left panel) and *RyR2*^*S/S*^ (d, left panel) in the presence of 0, 1 and 5 *μ*m flecainide. The right panels in (c) and (d) compare the maximum peak currents before and following withdrawal of flecainide. *denotes significant effects of flecainide or dantrolene. ^†^denotes significant differences between *RyR2*^*S/S*^ and *WT* genotypes at the same flecainide concentration.

**Figure 4:**
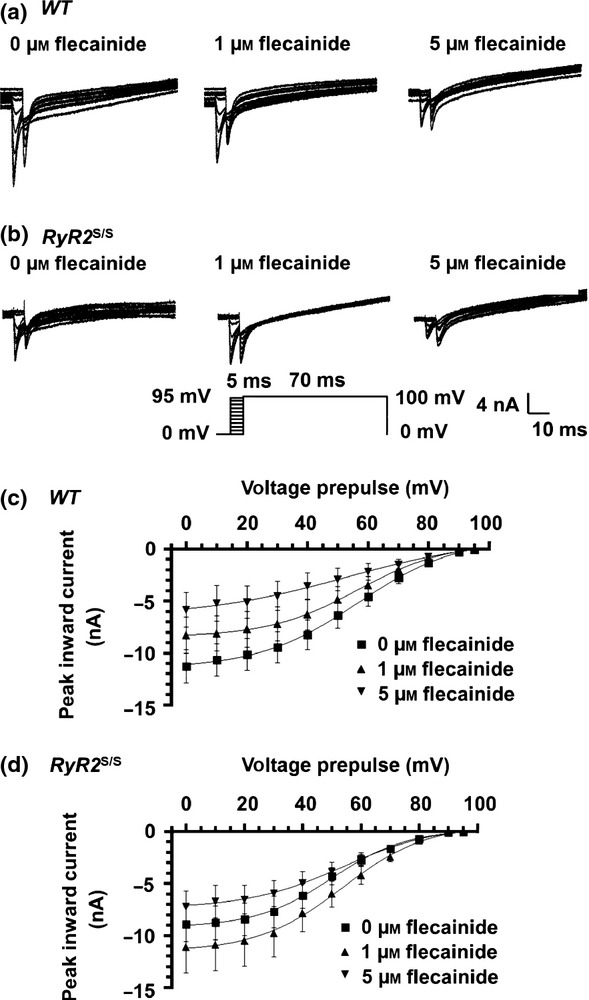
Paradoxical actions of flecainide on *I*_Na_ inactivation in *RyR2*^S/S^ and *WT*. Currents in response to successively incremented pre-pulse voltages from 0 to 90 mV, and finally 95 mV, followed by a test voltage excursion of 100 mV in voltage-clamped *WT* (a, *n* = 7) and *RyR2*^S/S^ (b, *n* = 6) left atria. (c, d) The dependence of peak *I*_Na_ upon pre-pulse voltage excursion fitted to Boltzmann functions for *WT* (c) and *RyR2*^S/S^ (d) in the presence of 0, 1 and 5 *μ*m flecainide. These experiments employed the same atria as in the experiments depicted in Figure3.

Activation and inactivation curves were obtained by plotting peak inward currents, *I*_Na,max_, against V (Figs3 and 4 respectively). These could be fitted to Boltzmann functions to provide empirical indications of maximum peak currents (*I*_Na,max_), steepness factors (*k*) describing their dependence upon voltage and the voltage excursions corresponding to half-maximal current (V*). Such optimizations were possible for all activation and inactivation data apart from *WT* atria studied in 5 *μ*m flecainide. Both protocols demonstrated that *RyR2*^S/S^ had a significantly reduced maximal inward Na^+^ current (*I*_Na(max)_) compared to *WT* (Fig.3, activation: *t =* 8.48; *P <* 0.001; Fig.4, inactivation: *t =* 8.42; *P <* 0.001), but, whereas 1 *μ*m flecainide reduced *I*_Na(max)_ in *WT* (activation: *t =* 12.53, *P <* 0.001, inactivation; *t =* 17.88, *P <* 0.001), it paradoxically *increased* such inward currents in *RyR2*^S/S^ (activation: *t =* 4.38, *P <* 0.01, inactivation; *t =* 8.84, *P <* 0.001). Five micromolar flecainide decreased *I*_Na(max)_ in both *RyR2*^S/S^ and *WT* (inactivation: *t =* 9.42, *P <* 0.001 and *t =* 11.47, *P <* 0.001 respectively).

Similarly contrasting effects on *I*_Na(max)_ were obtained with the specific RyR blocker dantrolene (10 *μ*m) (Fig.3, inset). Thus, in response to 80 mV voltage steps, dantrolene produced a net decrease in *I*_Na(max)_ in *WT* atria (−15.08 ± 1.68 vs. −10.89 ± 1.42, *n* = 7, *t =* 3.05; *P* < 0.05), but increased *I*_Na(max)_ in *RyR2* atria (−8.04 ± 1.19 vs. −11.54 ± 1.00, *n* = 7, *t* = 4.26; *P* < 0.01).

The *k* of the activation curves were indistinguishable between both genotypes and through all flecainide concentrations (Fig.3, *P* > 0.05). The *k* of the inactivation curves were similar, though smaller in the *RyR2*^S/S^ than the *WT* atria in the absence of flecainide (Fig.4, *t =* 2.79, *P <* 0.05). This difference was abolished by 1 *μ*m flecainide (*t =* 0.16, *P* > 0.05). Additional increases in flecainide concentration to 5 *μ*m further increased *k* in both *RyR2*^S/S^ and *WT* (*t =* 2.73, *P <* 0.05 and *t =* 1.86, *P* > 0.05) compared with 0 *μ*m flecainide, and compared with 1 *μ*m flecainide in *WT (t =* 2.32, *P* < 0.05).

Finally, V* of activation was consistently smaller in *RyR2*^S/S^ than in *WT* atria whether in 0 (*t =* 2.92; *P <* 0.05) or 1 *μ*m flecainide (*t =* 3.48; *P <* 0.01, Fig.3). Flecainide (1 *μ*m) decreased V* in *WT* (*t =* 3.39; *P <* 0.01), but not *RyR2*^S/S^ (*t =* 0.57, *P* > 0.05). The V* of inactivation was similarly reduced in untreated *RyR2*^S/S^ compared to *WT* (*t =* 5.75; *P <* 0.001, Fig.4). However, 1 *μ*m flecainide increased V* in *RyR2*^S/S^ (*t =* 2.87, *P <* 0.05) but not WT (*t =* 0, *P* > 0.05), with *RyR2*^S/S^ showing a smaller V* than *WT* (*t =* 3.02, *P <* 0.05). Increases in flecainide concentration to 5 *μ*m similarly increased V* in *RyR2*^S/S^ (*t =* 3.43, *P <* 0.01) relative to findings with 0 *μ*m flecainide. It left V* in *WT* close to that obtained at 0 *μ*m flecainide (*t =* 1.26, *P* > 0.05) as well as the corresponding result in the *RyR2*^S/S^ (*t =* 0.20; *P* > 0.05). Thus, both *k* and V* values in the activation and inactivation characteristics in the atria of *RyR2*^S/S^ and *WT* mice showed consistent patterns with the addition of 0, 1 and 5 *μ*m flecainide.

### Flecainide slows AP conduction in WT but not RyR2^S/S^ atria

**Figure 5:**
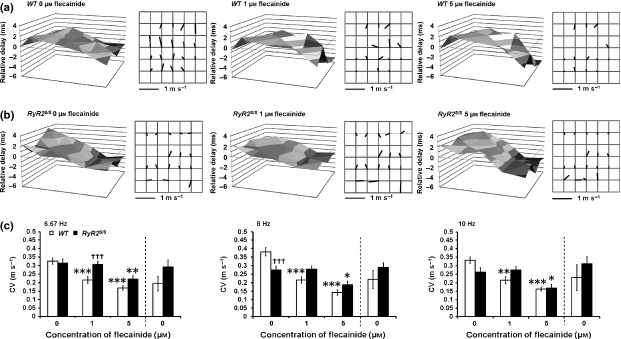
Paradoxical actions of flecainide on conduction velocities in *RyR2*^S/S^ and *WT*. Three-dimensional representations of local activation times (LATs) each accompanied by matrices representing the calculated velocity vectors in *WT* (a, *n* = 15) and *RyR2*^S/S^ hearts (b, *n* = 14) in 0, 1 and 5 *μ*m flecainide. Mean (±SEM) epicardial conduction velocities for *WT* (clear bars) and *RyR2*^S/S^ (black bars) in 0, 1, 5 and following subsequent return to 0 *μ*m flecainide during regular 6.67, 8 and 10 Hz pacing (c). *denotes a difference arising from use of 1 *μ*m flecainide within a genotype compared to the respective control (0 *μ*m flecainide). ^†^denotes a difference between *RyR2*^S/S^ and *WT* genotypes with the same concentrations of flecainide. In each case, single, double and triple symbols denote *P* < 0.05, *P* < 0.01 and *P* < 0.001 respectively.

Conduction velocities were determined by mapping LATs in *WT* and *RyR2*^*S/S*^ hearts before and following the addition of 1 and 5 *μ*m flecainide (Fig.5). CV progressively decreased with increasing flecainide concentrations in *WT*. In contrast, flecainide (1 *μ*m) did not affect CV in *RyR2*^*S/S*^. Nevertheless, 5 *μ*m did reduce CV in *RyR2*^S/S^ in common with the findings in *WT*. These findings applied to all pacing frequencies. Where washouts were performed, these confirmed at least a partial reversibility of flecainide's effects in the *WT*, and full reversibility in the *RyR2*^*S/S*^, at all the investigated pacing rates.

Two-way anova demonstrated significant effects of flecainide on CV at all pacing frequencies (6 Hz: *F* = 20.11, *P* < 0.001; 8 Hz: *F* = 25.72, *P* < 0.0001; 10 Hz: *F* = 13.88, *P* < 0.0001), while genotype differences were significant only at 6 Hz (6 Hz; *F* = 7.067, *P* = 0.0106, 8 Hz; *F* = 0.001364, *P* = 0.9707, 10 Hz; *F* = 0.004279, *P* = 0.9481). Nevertheless, the interaction between genotype and flecainide was significant at all frequencies (6 Hz: *F* = 3.456, *P* = 0.0396; 8 Hz: *F* = 8.383, *P* = 0.0006; 10 Hz: *F* = 3.375, *P* = 0.0433), indicating that the effect of flecainide is dependent upon both its concentration and genotype. Thus, a full pairwise comparison between all factors was permitted.

At 6 Hz pacing, *post hoc* tests demonstrated significant differences between *WT* and *RyR2*^S/S^ when both were treated with 1 *μ*m flecainide (*t* = 3.316, *P* < 0.001). One micromolar flecainide significantly reduced CV only in the *WT* (*t* = 3.943, *P* < 0.001), while 5 *μ*m reduced CV in both the *WT* (*t* = 5.471, *P* < 0.001) and *RyR2*^S/S^ (*t* = 3.343, *P* < 0.01).

At 8 Hz pacing, *post hoc* tests demonstrated that CV was significantly slower in the untreated *RyR2*^S/S^ relative to the untreated *WT* (*t* = 3.952, *P* < 0.001). Similar to 6 Hz, 1 *μ*m flecainide significantly reduced CV only in the *WT* (*t* = 5.604, *P* < 0.001) while 5 *μ*m reduced CV in both the *WT* (*t* = 7.206, *P* < 0.001) and *RyR2*^S/S^ (*t* = 2.826, *P* < 0.05).

At 10 Hz pacing, *post hoc* tests demonstrated that flecainide significantly reduced CV only in the *WT* at 1 *μ*m (*t* = 3.748, *P* < 0.01) and in both the *WT* and the *RyR2*^S/S^ at 5 *μ*m (*t* = 4.179, *P* < 0.001 and *t* = 2.824, *P* < 0.05 respectively).

### RyR2^S/S^ AERP increases with flecainide treatment, while WT AERP remains unchanged

**Figure 6:**
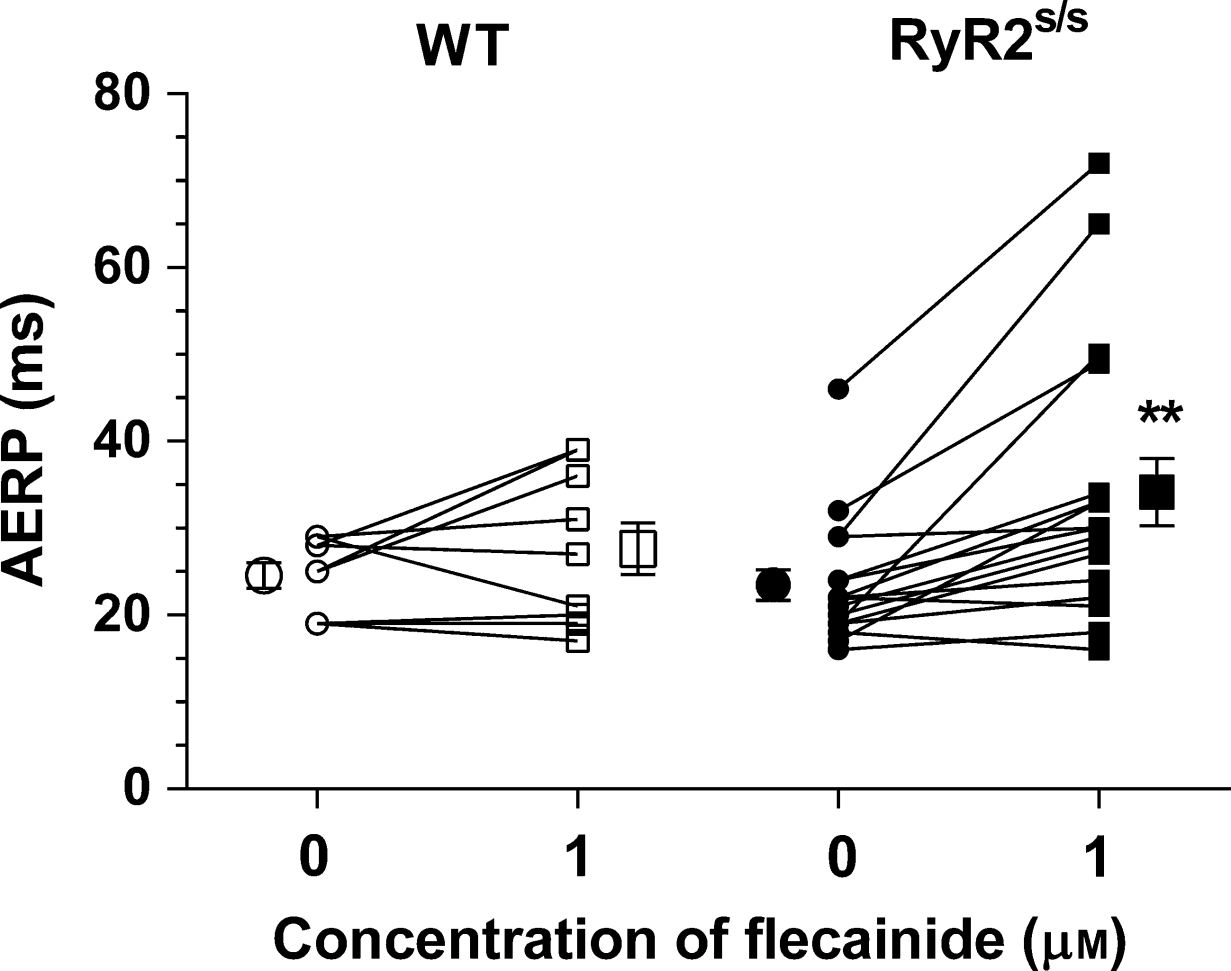
Paradoxical actions of flecainide on AERP in *RyR*^S/S^ and *WT*. Individual paired and mean (±SEM) AERPs in 0 and 1 *μ*m flecainide for *WT* (*n* = 9) and *RyR2*^S/S^ (*n* = 17) hearts. ** denotes a difference (*P* < 0.01) arising from use of 1 *μ*m flecainide within a genotype compared to the respective control (0 *μ*m flecainide).

Individual and mean (±SEM) AERPs from *WT* and *RyR2*^S/S^ before and following addition of 1 *μ*m flecainide are shown in Figure6 and Table 1. To a first approximation, AERP would be expected to depend mainly on action potential duration (APD) and Na^+^ channel availability in the final repolarization phase. However, two-way anova empirically demonstrated that flecainide increased AERP only with flecainide intervention (*F* = 4.761, *P <* 0.05). It did so with application of flecainide (1 *μ*m) in *RyR2*^S/S^ (*t* = 3.82; *P <* 0.01) but not in *WT*. Basal AERP values were indistinguishable between untreated *WT* and *RyR2*^S/S^ in agreement with previous reports (King *et al*. 2013c). Previous studies suggest that these changes likely take place in an absence of changes in APD in the *RyR2*^*S/S*^ system (King *et al*. 2013c). Furthermore, it has been shown in the *Scn5a*^*+/−*^ system, in which there was a loss of Na^+^ channel function, that flecainide produced a shortening of the APD in the face of a lengthening VERP (Martin *et al*. 2011).

### Action potential wavelengths correlate with arrhythmic incidence in both RyR2^*S/S*^ and WT

Two-way anova demonstrated strong interactions (*P =* 0.0021; *F* = 13.72) between the effects of flecainide and genotype upon *λ*, indicating that the effect of flecainide is different in *WT* compared to *RyR2*^S/S^. Flecainide decreased *λ* in *WT* (*t* = 2.39, *P <* 0.05) while increasing it in *RyR2*^S/S^ atria (*t* = 2.42, *P <* 0.05).

**Figure 7:**
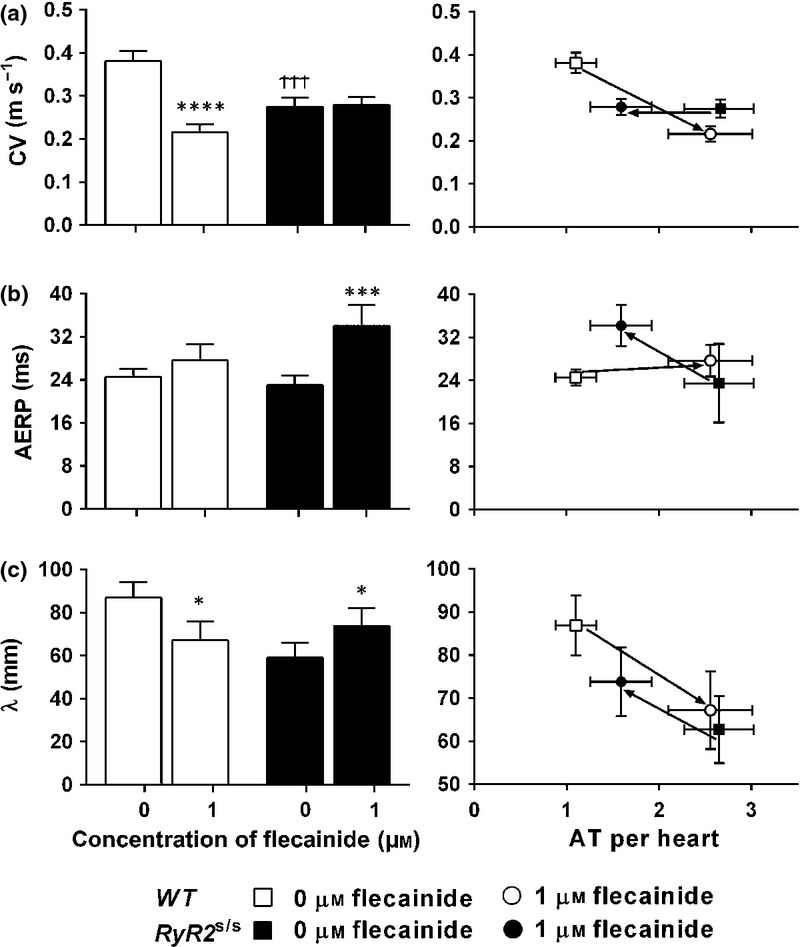
Paradoxical actions of flecainide on CV, AERP and *λ* and their correlations with arrhythmic incidence. Left panels: comparison of CV (a), AERP (b) and *λ* (c) in *WT* (open bars, *n* = 8) and *RyR2*^*S/S*^ (filled bars, *n* = 10) hearts in 0 and 1 *μ*m flecainide. These are correlated with incidences of atrial tachyarrhythmias (AT) (a–c, right panels). * denotes a difference arising from use of 1 *μ*m flecainide within a genotype compared to the respective control (0 *μ*m flecainide). ^†^ denotes a difference between *RyR2*^*S/S*^ and *WT* genotypes under the same concentration of flecainide. In each case, single, double and triple symbols denote *P* < 0.05, *P* < 0.01 and *P* < 0.001 respectively.

We then correlated CV, AERP and *λ* with arrhythmic incidences before and following application of 1 *μ*m flecainide (Fig.7). As indicated above, flecainide significantly reduced CV in *WT* but not *RyR2*^S/S^, directly correlating with the increased arrhythmic incidence in *WT* but not the decreased incidence of arrhythmia in *RyR2*^S/S^ (Fig.7A). In contrast, flecainide significantly increased AERP in *RyR2*^S/S^ but not *WT* atria directly correlating with the decreased arrhythmic incidences in *RyR2*^S/S^ but not the increased arrhythmic incidences in *WT* (Fig.7B). However, flecainide decreased *λ* in *WT* but increased *λ* in *RyR2*^S/S^ (Fig.7C)***.*** In contrast to CV and AERP, changes in *λ* therefore correlated with alterations in arrhythmia in both *RyR2*^S/S^ and *WT*. This implicates *λ* as the primary predictor for arrhythmic incidences rather than either CV or AERP alone, in both *RyR2*^S/S^ and *WT*.

## Discussion

The present study demonstrates a novel paradoxical effect of the *I*_Na_ blocker flecainide on arrhythmic incidence and *I*_Na_ in *RyR2-P2328S* and *WT* atria. It follows directly from evidence for its anti-arrhythmic effects in human CPVT (Pott *et al*. 2011, van der Werf *et al*. 2011, Watanabe *et al*. 2013). Its findings complement a recent report that the alternative class Ic anti-arrhythmic agent, propafenone, similarly exerted anti-arrhythmic actions during atrial fibrillation in a CSQ2^−/−^ model of CPVT (Faggioni *et al*. 2014). Although *I*_Na_ and CV were not measured in that latter study, the two reports converge upon common arrhythmic mechanisms through differing measurements and experimental systems. It also reconciles several previous studies at the cellular as opposed to tissue level. These attributed the anti-arrhythmic effects of flecainide to a range of factors. First, flecainide was suggested to reduce triggered activity arising from DADs (Liu *et al*. 2011). This could result from direct actions inhibiting spontaneous RyR2-mediated SR Ca^2+^ release implicated in such DADs: flecainide blocks RyR2-Ca^2+^ release channel open states, thereby reducing Ca^2+^ wave frequency in *CSQ2*^*−/−*^ mice and rat myocytes (Watanabe *et al*. 2009, Hilliard *et al*. 2010, Galimberti & Knollmann 2011). However, a subsequent study reported that whereas flecainide prevented isoproterenol-induced CPVT, it did not exert major effects on Ca^2+^ homeostasis in *RyR2-R4496C* hearts (Liu *et al*. 2011). This suggested that flecainide increases the threshold for triggered activity by directly inhibiting Na_v_1.5 function (Liu *et al*. 2011). Second, reductions in Na^+^ entry could reduce intracellular [Na^+^], thereby increasing forward-mode NCX activity, in turn reducing intracellular [Ca^2+^] (Sikkel *et al*. 2013). The alternative *I*_Na_ blockers, tetrodotoxin, propafenone or lidocaine similarly reduced Ca^2+^ spark and wave frequency, and wave velocity in *WT* rat myocytes, doing so only before *I*_Na_ inactivation brought about by alterations in holding voltage. Flecainide also increased NCX-mediated Ca^2+^ efflux, an effect reversed by reducing extracellular [Na^+^] (Sikkel *et al*. 2013).

However, recent findings also associated both catecholamine-induced ventricular arrhythmia (Zhang *et al*. 2013b) and atrial arrhythmogenesis with reductions in CV also associated with *RyR2*^*S/S*^ (King *et al*. 2013a,b,c). *RyR2*^S/S^ atria showed reduced *I*_Na_ compared to *WT*. Increased AF susceptibility in association with conduction abnormalities has also been observed in other models of altered Ca^2+^ homeostasis, including murine CREM-IbΔC-X AF (Li *et al*. 2014) and *CSQ2*^−/−^ hearts (Glukhov *et al*. 2013). In *WT*, elevating extracellular Ca^2+^ and manipulating cellular Ca^2+^ homeostasis using caffeine or cyclopiazonic acid acutely replicated these effects (Zhang *et al*. 2011).

These findings suggest that RyR2-mediated Ca^2+^ release in *RyR2*^S/S^ results in inhibition of *I*_Na_ reducing CV, thus producing a re-entrant, arrhythmic substrate. Inhibition of RyR2-mediated Ca^2+^ release by flecainide should then paradoxically restore *I*_Na_ and rescue both the compromised CV and arrhythmic phenotype. Our findings confirm this prediction: untreated murine *RyR2*^S/S^ atria were more arrhythmic than *WT*, confirming recent findings (King *et al*. 2013b,c), and at the cellular level showed reduced *I*_Na_ compared to the corresponding *WT*. Flecainide (1 *μ*m) was anti-arrhythmic in *RyR2*^S/S^ despite being pro-arrhythmic in *WT*. These findings were concordant with findings at the cellular level in which untreated *RyR2*^S/S^ showed reduced *I*_Na_ compared to the corresponding *WT*. Flecainide then reduced *I*_Na_ in *WT* while increasing it in *RyR2*^S/S^. The use of an alternative more specific RyR blocker, dantrolene (10 *μ*m), similarly reduced *I*_Na_ in *WT* atria while increasing it in *RyR2*^S/S^ atria. Dantrolene has previously been shown to reduce Ca^2+^ spark frequency and arrhythmogenicity in induced pluripotent stem cells derived from a CPVT patient carrying a *RYR2 S406L* mutation (Jung *et al*. 2012). The pro-arrhythmic action of flecainide in the *WT* may appear surprising due to its clinical utility for atrial tachycardia without structural abnormality. However, flecainide has proved pro-arrhythmic in various models (Brugada *et al*. 1991, Stokoe *et al*. 2007) and most notoriously in the cardiac arrhythmia suppression trial (CAST 1989). It has been proposed that this may result from effects on cardiac repolarization, and indeed, there is evidence for reduced *I*_Kr_ in cardiac cells (Follmer & Colatsky 1990, Wang *et al*. 1996), prolonged QT interval in human patients (Katritsis *et al*. 1995, Sarubbi *et al*. 1998), and repolarization abnormalities and increased arrhythmic incidences in perfused guinea-pig hearts (Osadchii 2012). The present results additionally suggest that reduction in *I*_Na_*,* CV and *λ* may contribute to the pro-arrhythmic effects of flecainide. Thus, at the tissue level, untreated *RyR2*^S/S^ showed reduced CVs compared to *WT*, despite similar AERPs. Flecainide decreased CV but conserved AERP in *WT*, whereas it spared CV and increased AERP in *RyR2*^S/S^. Nevertheless, *λ* derived from the product CV x AERP correlated directly with arrhythmic tendency in both the *RyR2*^S/S^ and *WT* under conditions of either 0 or 1 *μ*m flecainide.

These electrophysiological findings in intact atria are compatible with previous evidence for interactions between Ca^2+^ homeostasis and Na_v_1.5 expression and function in *WT* myocytes at the cellular level. Increases in pipette Ca^2+^ concentration reduced *I*_Na_ density and (d*V*/d*t*)_max_ in patch-clamped *WT* myocytes (Casini *et al*. 2009). The Ca^2+^ channel blocker verapamil and the Ca^2+^ ionophore calcimycin, respectively, increased and decreased Na_v_1.5 mRNA and Na_v_1.5 protein expression in rat cardiomyocytes (Offord & Catterall 1989, Taouis *et al*. 1991, Duff *et al*. 1992). Increased extracellular [Ca^2+^] and BAPTA-AM, respectively, expected to increase and decrease intracellular [Ca^2+^] and correspondingly increased and decreased *I*_Na_ density in cultured neonatal rat myocytes (Chiamvimonvat *et al*. 1995).

The findings also agree with previous evidence for mechanisms linking Ca^2+^ homeostasis to Na_v_1.5 at the molecular level. Na_v_1.5 is a major calmodulin kinase II (CaMKII) target. Such phosphorylation shifts the voltage dependence of inactivation to negative potentials without affecting channel activation. This slows recovery from inactivation, enhances Na_v_1.5 transitions into slower forms of inactivation and increases late *I*_Na_ (Wagner *et al*. 2011, Grandi & Herren 2014). However, in the present study, although *RyR2*^*S/S*^ was associated with a negative shift in inactivation, activation properties were similarly affected. *RyR2*^*S/S*^ showed a similar AERP as *WT* in the absence of flecainide.

The findings together demonstrate contrasting anti- and pro-arrhythmic actions of the Na_v_1.5 channel blocker flecainide in murine *RyR2*^S/S^ and *WT* atria respectively. They attribute these to corresponding changes in *I*_Na_, *λ* and therefore arrhythmic substrate while not excluding involvement of triggered activity in initiating arrhythmia with either genotype. This could involve a mechanism consistent with previously reported suggestions at the cellular level of interactions between cellular Ca^2+^ homeostasis and Na_v_1.5 function.
